# Association Between Citations, Altmetrics, and Article Views in Pediatric Research

**DOI:** 10.1001/jamanetworkopen.2020.10784

**Published:** 2020-07-20

**Authors:** Andrew J. Giustini, David M. Axelrod, Brian P. Lucas, Alan R. Schroeder

**Affiliations:** 1 Department of Anesthesiology, Stanford University School of Medicine, Stanford, California; 2 Department of Pediatrics, Stanford University School of Medicine, Stanford, California; 3 Dartmouth Institute for Health Policy and Clinical Practice, Hanover, New Hampshire; 4 Department of Medicine, Geisel School of Medicine at Dartmouth, Hanover, New Hampshire; 5 White River Junction Veterans Affairs Hospital, White River Junction, Virginia

## Abstract

This cross-sectional study examines the association between article citations, Altmetric attention scores, and cumulative page views of pediatric research articles from 4 high-impact medical journals.

## Introduction

Citations are used to assess the impact of scientific authors, articles, and journals but may not fully reflect how they affect journal readership or the public. Newer metrics include article views and the Altmetric attention score (AAS),^1^ which uses an algorithm to weight mentions of an article in social platforms such as Twitter and Facebook and in the news media. Prior investigations have shown only a modest association between these newer metrics and article citations, but none have assessed the pediatric literature, which may have a different readership.^1,2,3,4^ The primary objective of this study was to examine the association between traditional metrics (citations), the AAS, and views of pediatric articles from 4 high-impact journals. Strong correlation would imply that citations-based metrics indicate popular engagement in scientific articles.

## Methods

We examined all pediatric articles published in 2014—to allow sufficient time for citations to accrue—from 2 general pediatric journals (*Pediatrics* and *JAMA Pediatrics*) and 2 general medical journals (*JAMA* and the *New England Journal of Medicine*). Only articles tagged as *pediatrics* in *JAMA* and the *New England Journal of Medicine* were reviewed. All data were obtained between April and May 2018. We excluded supplementary issues of journals as well as news, videos, pictures, letters, errata, patient handouts, republished articles, calls for papers, and retracted articles.

Citations data were obtained from Web of Science (Clarivate), and the AAS and total cumulative page views were recorded from the journal sites. We communicated with each journal’s editorial office and confirmed that the sum of PDF downloads and page views was provided by each. We classified articles as *original research*, *clinical practice guidelines*, and *other* (predominantly editorials and reviews, not all of which were peer-reviewed).

Spearman rank correlation coefficients were calculated to compare the ranks of the 3 metrics. A Kruskal-Wallis test was used to compare metrics between research articles, practice guidelines, and other types of articles. Statistical analysis was done using R, version 3.3.1 (R Foundation for Statistical Computing) and the RStudio interface, version 1.0.143 (RStudio Inc).

## Results

Of 1162 included articles, 686 were classified as original research, 40 as clinical practice guidelines, and 436 as other. The correlation (95% CI) between ranks was 0.69 (0.66-0.72) for citations and page views, 0.53 (0.49-0.57) for citations and the AAS, and 0.53 (0.49-0.57) for page views and the AAS. Similar correlations were found when comparing actual citations rather than ranks (data not shown).

**Figure 1.**
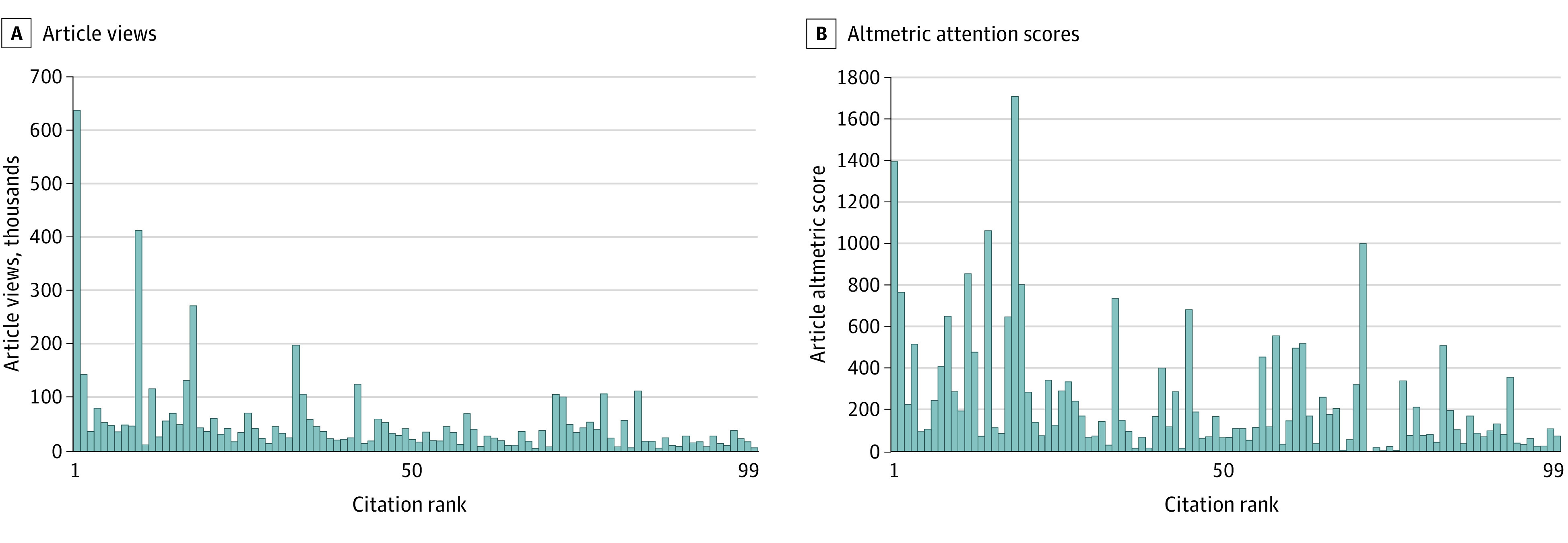
Article Views and Altmetric Attention Scores for the Top 100 Articles Ranked by Number of Citations The most frequently cited article is represented by 1.

**Figure 2.**
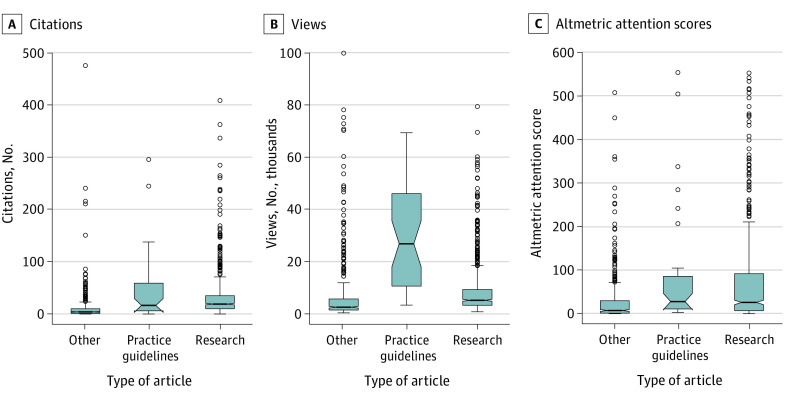
Citations, Views, and Altmetric Attention Scores by Article Type *Research* denotes any hypothesis-driven research article; *other* denotes any other article type. The horizontal bar inside the boxes indicates the median; lower and upper ends of the boxes, the first and third quartiles of the data; whiskers, the values within 1.5 times the interquartile range from the upper or lower quartile; open circles, outliers; and notches, 95% CIs.

Figure 1 shows the AAS and page view ranks for the 100 most-cited articles in 2014. Original research articles were more frequently cited than articles classified as other, and clinical practice guidelines were the most frequently viewed of any article type (Figure 2).

## Discussion

The results show only modest correlation between article citations and newer measures of journal impact (page views and the AAS) in pediatric articles from 4 journals. Citations may not fully convey an article’s impact. The AAS and page views may provide a different perspective on an article’s immediate impact and should be considered when evaluating the impact of an article.

This investigation had several limitations. Citations, views, and the AAS are all cumulative metrics, but the AAS tends to accumulate most of its value immediately after an article is published and may, uniquely, decrease over time^5^; therefore, the AAS may be an indicator of an article’s initial impact. Thus, this study compared snapshots in time of 3 dynamic processes. We examined only 4 journals, which were selected on the basis of their impact factor and the immediate online availability of all 3 metrics. Finally, page views do not capture print readership of articles.

In summary, citations may not fully capture the impact of scientific work. Newer metrics such as the AAS and page views should be considered when determining the overall impact of publications.

## References

[1] Thelwall M, Haustein S, Larivière V, Sugimoto CR Do altmetrics work? Twitter and ten other social web services. PLoS One. 2013;8(5):e64841-e64847. doi:10.1371/journal.pone.0064841 23724101 PMC3665624

[2] Rosenkrantz AB, Ayoola A, Singh K, Duszak R Jr Alternative metrics (“Altmetrics”) for assessing article impact in popular general radiology journals. Acad Radiol. 2017;24(7):891-897. doi:10.1016/j.acra.2016.11.019 28256440

[3] Fox CS, Bonaca MA, Ryan JJ, Massaro JM, Barry K, Loscalzo J A randomized trial of social media from Circulation. Circulation. 2015;131(1):28-33. doi:10.1161/CIRCULATIONAHA.114.013509 25406308 PMC10822683

[4] Nieder C, Dalhaug A, Aandahl G Correlation between article download and citation figures for highly accessed articles from five open access oncology journals. Springerplus. 2013;2(1):261. doi:10.1186/2193-1801-2-261 23853747 PMC3698439

[5] Why has the Altmetric Attention Score for my paper gone down? Altmetric. Updated January 9, 2019 Accessed July 14, 2019. https://help.altmetric.com/support/solutions/articles/6000108191-why-has-the-altmetric-attention-score-for-my-paper-gone-down-

